# Effects of 12 Months of Vitamin D Supplementation on Physical Fitness Levels in Postmenopausal Women with Type 2 Diabetes

**DOI:** 10.3390/jfmk6040087

**Published:** 2021-10-25

**Authors:** Claudio Melibeu Bentes, Pablo B. Costa, Monique Resende, Claudia Netto, Ingrid Dias, Anderson Luiz Bezerra da Silveira, Fabrizio Di Masi, Humberto Miranda, Lucas Monteiro de Carvalho, Lizanka Marinheiro

**Affiliations:** 1 Laboratory of Physiology and Human Performance, Department of Physical Education and Sports, Institute of Education, Federal Rural University of Rio de Janeiro, Seropedica 23890-000, RJ, Brazil; claudiomelibeu@ufrrj.br (C.M.B.); andersonsilveira@ufrrj.br (A.L.B.d.S.); fabriziomasi@ufrrj.br (F.D.M.); lucasmonteirodc@gmail.com (L.M.d.C.); 2 Graduate Program, Fernandes Figueira Institute, Oswaldo Cruz Foundation, Rio de Janeiro 22250-020, RJ, Brazil; moniqueres@gmail.com; 3 Department of Kinesiology, California State University, Fullerton, CA 92831-3547, USA; lizanka@globo.com; 4 Department of Biochemistry, School of Medicine and Surgery, Biomedical Institute, Federal University of the State of Rio de Janeiro (UNIRIO), Rio de Janeiro 20211-040, RJ, Brazil; claucnetto@gmail.com; 5 Physical Education Graduate Program, Department of Gymnastics, School of Physical Education and Sports, Federal University of Rio de Janeiro, Rio de Janeiro 21941-599, RJ, Brazil; ingriddias.ufrj@gmail.com (I.D.); humbertomiranda@eefd.ufrj.br (H.M.); 6 Physiological Sciences Multicenter Graduate Program, Department of Physiological Sciences, Health Sciences Center, Institute of Biological and Health Sciences, Federal Rural University of Rio de Janeiro, Seropedica 23890-000, RJ, Brazil; 7 Physical Education Graduate Program, Performance, Training, and Physical Exercise Laboratory, Department of Gymnastics, School of Physical Education and Sports, Federal University of Rio de Janeiro, Rio de Janeiro 21941-599, RJ, Brazil

**Keywords:** cholecalciferol, elderly women, functional capacity, metabolism, athletic performance, hyperglycemia

## Abstract

Introduction: It is common for postmenopausal women to receive a vitamin D supplementation prescription to assist in preventing future falls and to maintain bone health. However, the association between vitamin D supplementation and physical fitness components has not been studied in older women with diabetes. Objective: We examined the influence of 12 months of vitamin D supplementation on the components of physical fitness in postmenopausal women with type 2 diabetes mellitus (T2DM). Methods: Thirty-five postmenopausal women (62.48 ± 7.67 years; 154.6 ± 5.11 cm; 73.93 ± 15.43 kg; 31.13 ± 5.82 BMI) with a diagnosis of T2DM participated in this longitudinal study where participants were supplemented with 1000 IU/day of vitamin D over 12 months. Subjects performed fasting blood samples, anthropometric assessments, body composition, clinical exams, and physical tests at 6-month intervals (P0, P6, and P12). Results and Conclusion: Vitamin D supplementation alone was effective in postmenopausal women in increasing serum vitamin D levels, altering muscle strength levels, promoting improvements in muscle function, as well as preventing and controlling fragility caused by T2DM and aging.

## 1. Introduction

The growth of the elderly population is a worldwide phenomenon, and it is estimated that over the next 30 years, the proportion of people over 65 will double from 12% to 22–25% in the US and Canada [1]. Due to the increase in life expectancy, several diseases related to the climacteric period of women now present serious problems for public health [2]. Women tend to report dizziness, tiredness, night sweats, depression, hot flashes, sleep disorders, as well as cardiovascular diseases, osteoporosis, metabolic syndrome, and diabetes, among others [3].

The changes occurring in the transition between menopause and post menopause are physiological, but some may cause symptoms that worsen the quality of life and others that may increase the risk of various diseases [2]. Low levels of female sex steroids, regardless of cause, can lead to endocrine and functional disorders such as sexual dysfunction, loss of libido, altered levels of lipoproteins, and increased risk of obesity, cardiovascular diseases, and cardiometabolic diseases such as type 2 diabetes mellitus (T2D) [3].

T2D is the most prevalent form of diabetes worldwide, representing 90% of global cases, and projection is expected to rise to 300 million cases worldwide by 2025 [4]. Despite the constant campaign to promote lifestyle changes, such as dietary re-education and inclusion of physical activity for better metabolic control, these are difficult measures to achieve and maintain [5]. Similar to T2D, vitamin D deficiency is considered a worldwide epidemic with multiple implications for human health because of its role in various physiological systems [6]. However, several studies have already shown that normal and higher levels of vitamin D may reduce the incidence of non-vertebral fractures and hip fractures [6].

One of the age groups that suffers with comorbidities associated with hypovitaminosis D is postmenopausal women [6]. Several longitudinal studies have already demonstrated an association between hypovitaminosis D and the increased risk of chronic diseases such as cardiovascular diseases [7] and T2D [8]. Low levels of vitamin D and the development of T2D may be associated with the action of insulin resistance, increasing glucose intolerance [9]. In addition, in the postmenopausal period, it is common for women to receive a prescription for vitamin D supplementation as a method of prevention for future falls and maintenance of bone health. However, the association between vitamin D supplementation and physical fitness components has not been studied in the scientific literature in menopausal and diabetic women, although a few studies have investigated the effects of vitamin D supplementation on strength and muscular power gains [10].

In a recent review of the literature, Bentes et al. [11] reported the shortage of studies, and in a short review, the authors describe five longitudinal studies concluding that for vitamin D to have an effect on the postmenopausal age range, daily doses need to be greater than 1000 IU/day. Typically, these studies with vitamin D_3_ supplementation used the cholecalciferol form. Consequently, it is essential for longitudinal studies to investigate the effects of vitamin D supplementation on changes in physical fitness components in postmenopausal women with T2D, since this topic requires further investigation as it may represent an important resource in the control and treatment of fragility caused by aging. Therefore, the purpose of the present study was to examine the effects of vitamin D supplementation for 12 months on physical fitness in postmenopausal women with T2D.

## 2. Methods

### 2.1. Participants and Research Design

A longitudinal, paired clinical study, with a quasi-experimental characteristic, was performed in a period of 12 months. One hundred and ten women from the gynecology outpatient clinic, who were already postmenopausal and diagnosed with T2D, were recruited to participate in the study. After the invitation, the following exclusion criteria were applied: neurological problems that compromise balance and gait; patients who were supplementing with a dose of more than 1000 u/d of vitamin D (all types) continuously for more than three months; cognitive deficits, deformities in the upper and lower limbs, severe vision problems, dizziness symptoms, severe hearing loss, uncontrolled arterial hypertension, and postural hypotension, use of balance-compromising drugs (sedatives and anticonvulsants); excessive use of alcohol; obesity Grade III, liver diseases and nephropathies, use of glucocorticoids, antiretroviral medication for HIV, hyperparathyroidism, hypercalcemia, lymphomas, granulomatous diseases, current neoplasm. The initial group for the study was 40 participants at the end of the recruitment and consent (Table 1).

### 2.2. Procedures

Participants who met the criteria and who signed the term during the invitation consultation were aware of the start and end dates of data collection and the amount of vitamin D that would be administered, as well as the procedures for the collection, where they should be fasting for 12 h and in light and comfortable clothes. The kits were delivered at the first visit with the amount of supplementation for three months with the daily amount of 1000/ud of vitamin D (cholecalciferol) in pill form and subjects were instructed to return after three months with the empty bottles to be replenished.

**Figure 1:**
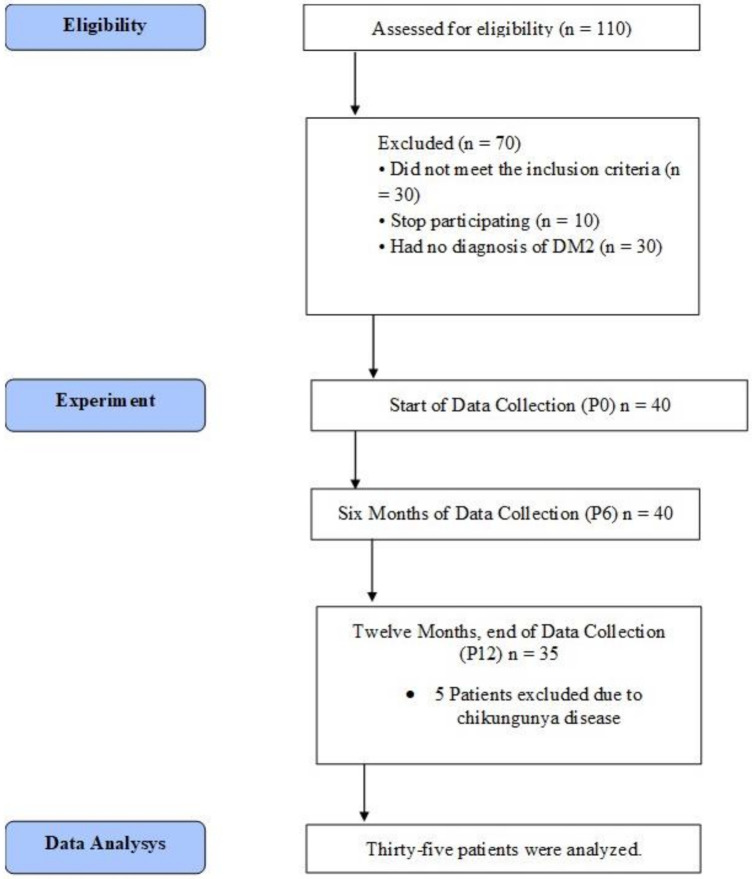
Data Collection Flowchart.

All physical tests were performed in random order. At the first visit (P0), the patient underwent blood collection, routine outpatient consultation, body composition analysis, and then received breakfast. After these initial tests, the subjects participated in the physical fitness tests. The routine outpatient consultation consisted of a complete medical examination and analysis of the patient’s health status. If necessary, the physician prescribed medication to control the diabetes with oral glucose-regulating drugs. The appointments for replacement of the vitamin D kits and routine laboratory examinations were rescheduled at three-month intervals. However, the procedures for analysis were only repeated with the 6-month intervals (P6 and P12). In addition, they were performed by the same investigators. Thus, the measures for the analysis of the outcome were performed in the following moments: pre-experiment (P0), six months after (P6), and 12 months after (P12) (Figure 1).

### 2.3. Physical Tests

Handgrip strength was measured using a hydraulic grip strength dynamometer (Jamar Hydraulic Hand Dynamometer Model J00105, Lafayette Instrument Company, La Fayette, IL, USA). Grip strength was measured three times using the dominant hand while the subject was in a seated position, shoulder adducted and neutrally rotated, elbow flexed at 90° with the forearm in neutral position, with a 1 min rest period between attempts. The highest recorded value of three attempts was used for analyses. Relative strength was calculated with the equation: handgrip strength (kg) ÷ body mass (kg), as suggested by Prestes and Tibana [12].

The timed up and go (TUG) test was used to examine functional mobility, muscle function, walking speed, and dynamic balance [13]. This test involves the time taken to rise from a chair, walk 3 m, turn around a marker, walk back to the chair, and sit down [13].

The arm curl test was used to measure the upper body local muscle endurance [14]. Subjects performed seated biceps curls without bending the trunk forward for 30 s with 2.3 kg dumbbells. The score used was the total number of arm curls.

The 30 s chair stand was used to measure lower limb local muscle endurance. The score equals the number of rises from a chair in 30 s with arms folded across the chest [14].

### 2.4. Anthropometry and Body Composition

Body height (BH) was measured with a stadiometer (Stadiometer Seca 208 Bodymeter), and waist, iliac, abdominal, and hip circumferences were measured with an anthropometric tape. In addition, body mass (BM), fat mass (FM), lean body mass, fat percentage, fat-free mass (FFM), and visceral fat area (VFA) were measured with an octopolar bioimpedance InBody 720 (Biospace, Seoul, Korea). The validity of this bioimpedance for body composition has been previously documented [15].

### 2.5. Blood Sample Analyses

Blood samples were collected after fasting for 12 h. The main outcome parameters were fasting plasma glucose and vitamin D (25-hydroxyvitamin D [25-OH D]). The serum fasting glucose was measured by using the enzymatic colorimetric (GOD-PAP) method. The serum vitamin D concentrations were assessed by chemiluminescent assay.

### 2.6. Statistical Analyses

Statistical analysis was initially performed using the Kolmogorov–Smirnov normality test and the homoscedasticity test (Bartlett criterion). All variables demonstrated normal distribution and homoscedasticity (*p* > 0.05). Repeated measures ANOVA was used to compare the means to verify possible differences in time (P0 vs. P6 vs. P12). In case of significant differences, a Bonferroni post hoc for analysis of multiple comparisons between variables was used (P0 vs. P6; P0 vs. 12; P6 vs. 12). The significance level adopted was *p* < 0.05 and IBM SPSS 24.0 software was used for all statistical analyses.

## 3. Results

### Primary Outcomes

**Figure 2:**
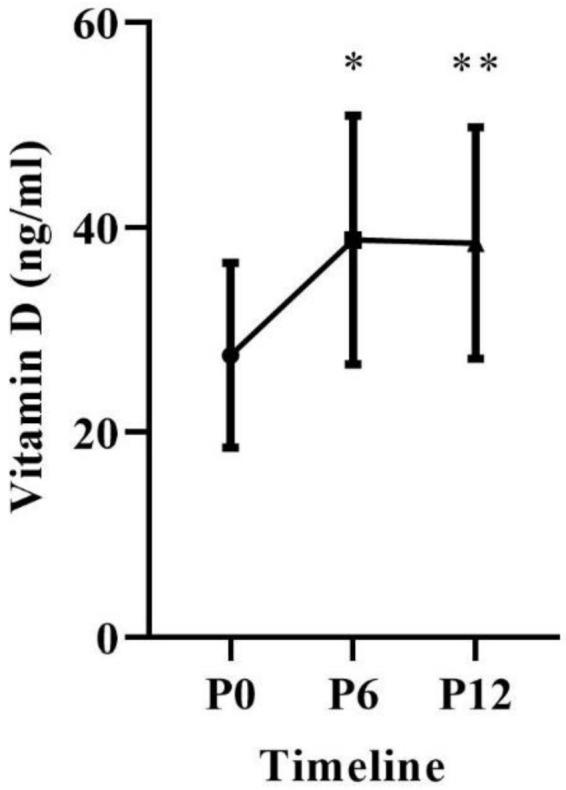
Blood concentration of 25 (OH) D over 12 months of supplementation. * Differences between P0 vs. P6, *p* = 0.0001. ** Differences between P0 vs. P12, *p* = 0.0001.

In the comparisons of repeated measurements (P0 vs. P6 vs. P12), the ANOVA results demonstrated patients had significant increases in vitamin D (*p* = 0.0001). In the multiple comparisons, there were gains between the measurements of P0 (27.5 ± 9.0; Limit) vs. P6 (38.8 ± 12.1; Acceptable); *p* = 0.0001 and a percentage increase of 48.39% (11.55 ng/mL), P0 (27.5 ± 9.0; Limit) vs. P12 (38.5 ± 11.3; Acceptable); *p* = 0.0001 and a percentage increase of 32.35% (6.14 ng/mL) (Figure 2).

In repeated measures comparisons (P0 vs. P6 vs. P12), the ANOVA results demonstrated the patients had significant changes in the following variables: handgrip strength (*p* = 0.0001), relative strength (*p* = 0.0001), arm curl test (*p* = 0.0002), and 30 s chair stand (*p* = 0.0001). Additionally, there were no differences in the following variables: timed up and go (*p* = 0.107), sit and reach (*p* = 0.625).

**Figure 3:**
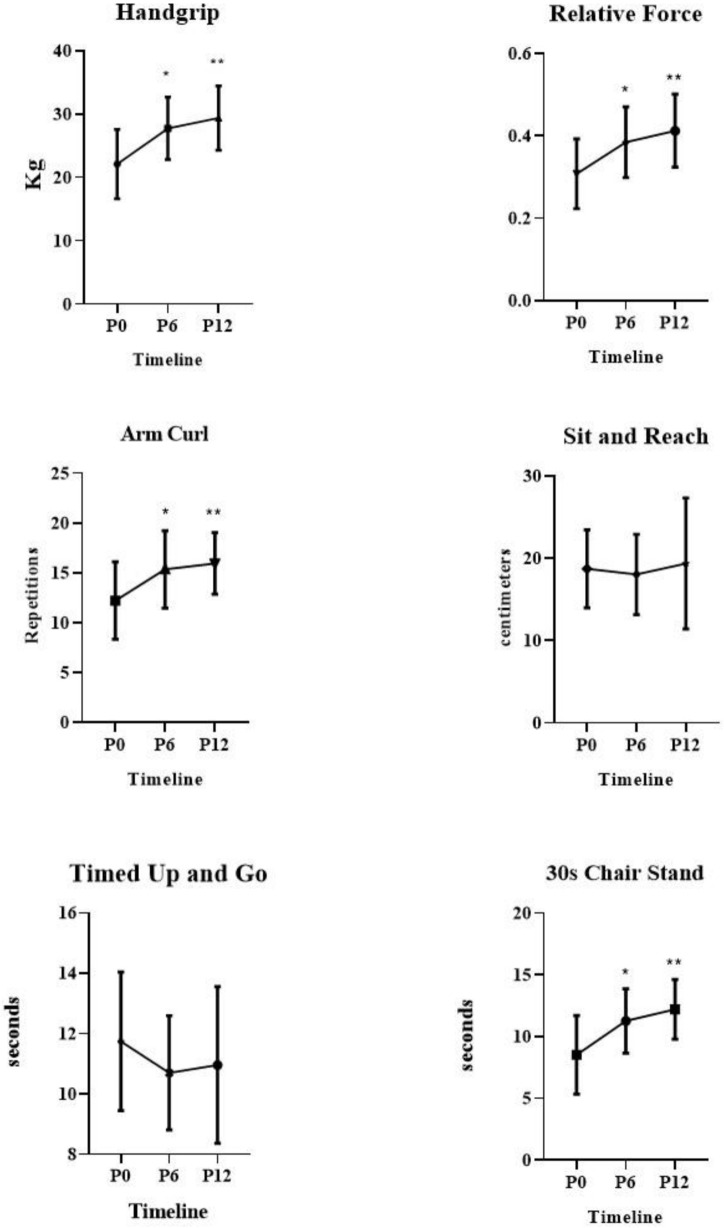
Physical Fitness results over 12-months of Vitamin D supplementation for elderly women. * Differences between P0 vs. P6, *p* = 0.0001, ** differences between P0 vs. P12, *p* = 0.0001.

In multiple comparisons, there were significant gains on handgrip strength between P0 (22.10 ± 5.47) vs. P6 (27.73 ± 4.94); *p* = 0.0001, P0 (22.10 ± 5.47) vs. P12 (29.37 ± 5.08); *p* = 0.0001. There were significant gains on relative strength between P0 (0.31 ± 0.08) vs. P6 (0.38 ± 0.09); *p* = 0.0001, P0 (0.31 ± 0.08) vs. P12 (0.41 ± 0.09); *p* = 0.0001. There were significant gains on arm curl test between P0 (12.20 ± 3.88) vs. P6 (15.35 ± 3.89); *p* = 0.0001, P0 (12.20 ± 3.88) vs. P12 (15.94 ± 3.09); *p* = 0.0001. There were significant gains on 30 s chair stand between P0 (8.53 ± 3.19) vs. P6 (11.26 ± 2.60); *p* = 0.0001, P0 (8.53 ± 3.19) vs. P12 (12.20 ± 2.41); *p* = 0.0001. Figure 3.

## 4. Secondary Outcomes

In repeated measures comparisons (P0 vs. P6 vs. P12), the ANOVA results demonstrated the patients had significant changes on waist/hip ratio (WHR), *p* = 0.04. In multiple comparisons, there were significant changes between P0 (0.93 ± 0.08) vs. P12 (0.88 ± 0.08); *p* = 0.041, Table 2.

## 5. Discussion

The main purpose of this study was to examine the influence of vitamin D supplementation for 12 months in postmenopausal women with T2DM on various components of physical fitness. Therefore, to follow the evolution of these outcomes, three time-points were tested over 12 months (P0, P6, and P12). Hypovitaminosis D is common in postmenopausal women. Possible causes of this condition include low sun exposure and reduced capacity to produce vitamin D, reduced renal function, lower absorption of vitamin D from the gastrointestinal tract, and use of multiple medications that may interfere with absorption and metabolism of this vitamin [16,17].

The main results in the present study demonstrated postmenopausal vitamin D supplementation alone, in addition to positively modifying serum levels of this limitrophe to desirable, and promoting significant increases in neuromuscular markers of physical fitness. In addition, the supplementation controlled the chronic loss of lean mass that is natural during aging and a characteristic of patients with T2D who also changed the distribution of body fat, altering indicators such as WHR.

Vitamin D availability in the body is assessed by measuring plasma concentrations of 25 (OH) D [18]. In participants of this study, serum concentrations were within the limitrophe classification in the beginning of data collection, which is below the desirable range. The International Osteoporosis Foundation and Endocrinology Society recommendations based on two meta-analyses suggest concentrations greater than 24 ng/mL to reduce fall rates [19] and above 30 ng/mL to reduce fracture rates [20]

In the present study, patients achieved the desirable classification (above 32 ng/mL) within 6 months and maintained it after 12 months with daily vitamin D supplementation (1000 IU/day). In any case, the population investigated in this clinical study consisted of postmenopausal women with T2DM who have this recommendation because they have risk factors for hypovitaminosis D [21].

Reduced vitamin D blood concentrations are associated with changes in muscle function [12]. Vitamin D receptors (VDR) are found in muscle tissue and are involved in activating muscle protein synthesis, meaning it is an important maintainer and important agent for muscle hypertrophy [22].

One study has shown vitamin D supplementation was able to increase serum VDR concentration by 30% and the amount of muscle fibers by 10% [23]. The main change induced by vitamin D deficiency is fast-twitch type 2 muscle atrophy, which is the first to be recruited during postural balance recovery, a fact that may explain the inverse association between serum levels of 25 (OH) D and falls [19].

The results of the present study support the data reported in the literature that during the follow-up period, as serum 25 (OH) D levels increased, participants had significant increases in muscle function at all times compared to P0. The most plausible explanation for this increase is in the study of Annweiler et al. [24], where the authors state that vitamin D supplementation can alter the oxidative capacity of muscle by improving muscle function.

There were significant increases in all physical fitness variables measured (handgrip, relative strength, arm curl test, 30 s chair stand) at moment P6 and P12. In a recent brief review, Bentes et al. [12] reported that only five studies to date have examined the influence of vitamin D supplementation alone on physical fitness markers; however, only three of the five studies using dosages at or above 1000 IU/day caused significant increases in strength gains, and this was the same dosage used in the present study during 12 months of follow-up.

Corroborating with our results, Zhu et al. [25] examined vitamin D supplementation on muscle strength and mobility in 302 elderly women (70–90 years) with 25 (OH) D insufficiency. The intervention group received doses of 1000 IU/day and the results show that based on baseline values, supplementation was able to increase muscle strength and muscle function in elderly women with vitamin D deficiency. Cangussu et al. [16] evaluated the daily supplementation of 1000 IU in 160 postmenopausal women, and the results demonstrated the intervention group obtained significant gains in vitamin D and consequently, significant increases in lower and upper limb muscle strength in addition to the control of lean mass loss. Similarly, the study by Anek et al. [26] investigated the effects of four weeks of vitamin D supplementation (20,000 IU/week) on markers of bone mass, muscle strength, and balance in 52 postmenopausal women (45–55 years). Results showed that after four weeks there were significant improvements in physical fitness.

In addition to the aforementioned fitness measures, as a secondary outcome, anthropometric measurements and bioimpedance were performed to assess body composition. Only the WHR measurement showed significant improvements. In contrast, during the 12 months of follow-up, there was maintenance of body mass, lean mass, and fat without any significant changes. This can be seen as a fundamental positive factor in the aging process, and an important factor in women with T2DM because of the acceleration in the process of muscle loss due to the pathological feature of the disease [27].

Nevertheless, WHR decreased by 4.08% in 12 months. The explanation lies in the process of transferring body fat. In the postmenopausal process, it is common for women to present changes in the postmenopausal process, it is usual for women to show changes in the abdominal/waist fat accumulation, with decreasing fat layer thickness in the hip and increasing in the visceral region [28]. Unexpectedly, in the present study vitamin D supplementation over the 12 months maintained body mass and body fat but presented significant decreases in WHR.

The strengths of this assay are serum vitamin D (25 (OH) D) measurements with the chemiluminescent assay that is most sensitive for detecting plasma vitamin D [29]. In addition, the functional measures of the present study have practical characteristics and fulfill the participants’ daily functions, providing more accurate information on the analysis of variables related to muscle function. The type of population studied (i.e., postmenopausal women with T2DM in an age group under 65 years) demonstrates a strength for the study.

Our most important limitation was not being able to form a control group, as the hospital ethics committee did not allow the formation of a placebo condition in this age category due to the high number of studies already suggesting the benefits for preventing falls and improving bone health. Hence, we highlight the sample size of 40 participants, although the sample size calculation showed a power of 93%, enhancing the external validity of the study. In addition, there could have been a memory bias due to the long period between consultations. However, the investigators performed biweekly calls and quarterly appointments for vitamin replacement and recall of test procedures. Furthermore, the selection bias is associated with admission of patients who were already part of the female endocrinology service which can be assumed to be periodically seen by medical professionals and had constant access to general health care. Another limitation is related to the control of whether the patients used the vitamin D doses, although participants were given biweekly instructions and returned empty bottles indicating total consumption of the product. Lastly, researchers did not have nutrition control of the participants’ diets and diet was not monitored. Thus, even though blood tests on P6 and P12 showed increases in serum vitamin D levels, the potential confounding variable of increasing serum vitamin D levels from diet intake alone cannot be overlooked.

## 6. Conclusions

In conclusion, vitamin D supplementation appeared to be effective in postmenopausal women to increase serum vitamin D levels, hence significantly altering muscle strength levels, and promoting improvements in muscle function, which can help control comorbidities associated with T2D and aging. In addition, serum vitamin D increased significantly, decreasing the WHR, demonstrating that it may be effective in redistributing body fat caused by menopause. Therefore, for 12 months patients maintained body mass, lean and fat mass, and increased muscle strength. Thus, it may be an excellent strategy for women in this age group who do not have any contraindication to supplement with this vitamin. Although the results demonstrate important benefits for postmenopausal diabetic women, the reduced sample size may limit external inferences. Nevertheless, we can consider these important findings as preliminary evidence of potential benefits of vitamin D supplementation for older women with diabetes.

## Figures and Tables

**Table 1:** Sample Characteristics.

	Mean	±	SD	K-S
Age (years)	62.48	±	7.67	0.067
Height (cm)	154.6	±	5.11	0.798
Body mass (kg)	73.93	±	15.43	0.052
Waist circumference (cm)	96.99	±	14.25	0.636
Abdominal circumference (cm)	98.46	±	11.54	0.666
Iliac circumference (cm)	101.42	±	12.24	0.545
Hip circumference (cm)	104.05	±	10.20	0.195
Waist/hip ratio	0.93	±	0.08	0.336
BMI (kg/m^2^)	31.13	±	5.82	0.404
Lean body mass (kg)	22.29	±	3.23	0.162
Fat mass (kg)	33.17	±	10.87	0.355
Fat percentage (%)	43.62	±	6.36	0.269
Visceral fat area (cm^2^)	124.36	±	31.06	0.248
Resting Metabolic Rate (kcal/d)	1259.54	±	116.20	0.112
Vitamin D status (ng/mL)	27.47	±	8.98	0.073
Fasting glucose (mg/dL)	144.65	±	55.99	0.092

Legend: K–S: Kolmogorov–Smirnov normality test. SD—Standard Deviation.

**Table 2:** Secondary Outcomes results.

Variable	Timeline	Mean	±	Standard Deviation	Confidence Interval 95%	
					**Lower**	**Higher**
Age (years)	P0	62.64	±	7.64	60.4	65.1
	P6	63.02	±	7.68	60.6	65.6
	P12	63.05	±	7.30	60.6	65.6
Body Mass (Kg)	P0	73.80	±	15.26	69.3	78.6
	P6	74.03	±	14.74	69.5	78.6
	P12	73.02	±	14.17	68.7	77.8
Waist circumference (cm)	P0	96.93	±	14.07	92.9	101.1
	P6	94.46	±	13.41	90.2	98.6
	P12	92.91	±	14.02	88.6	97.6
Abdominal circumference (cm)	P0	98.41	±	11.39	95.2	101.9
	P6	98.28	±	12.15	94.6	102.1
	P12	98.64	±	11.92	94.8	102.5
Iliac circumference (cm)	P0	101.68	±	12.19	98.2	105.6
	P6	101.82	±	11.05	98.4	105.4
	P12	103.80	±	11.56	100.1	107.5
Hip circumference (cm)	P0	104.13	±	10.07	101.1	107.3
	P6	104.60	±	9.13	101.7	107.5
	P12	104.85	±	9.68	101.7	108.0
WHR	P0	0.93	±	0.08	0.9	1.0
	P6	0.90	±	0.08	0.9	0.9
	P12	0.88 *	±	0.08	0.9	0.9
Lean body mass (kg)	P0	31.20	±	5.76	29.4	33.1
	P6	31.07	±	5.72	29.3	32.8
	P12	30.72	±	5.61	29.0	32.7
Bone Mass	P0	2.34	±	0.29	2.3	2.4
	P6	2.32	±	0.29	2.2	2.4
	P12	2.31	±	0.28	2.2	2.4
Lean body mass	P0	22.17	±	3.28	21.2	23.2
	P6	22.53	±	7.53	20.4	25.2
	P12	21.71	±	3.25	20.7	22.8
Fat mass	P0	33.24	±	10.74	30.2	36.5
	P6	33.29	±	10.57	30.0	36.7
	P12	32.80	±	10.25	29.7	36.3
Fat percentage (%)	P0	43.84	±	6.42	41.9	45.9
	P6	44.35	±	6.80	42.3	46.3
	P12	44.05	±	6.56	41.7	46.2
Visceral fat area	P0	124.09	±	31.42	114.7	133.6
	P6	123.26	±	29.43	114.5	132.5
	P12	123.39	±	28.95	114.4	133.5
Rest Metabolic Rate	P0	1255.10	±	118.08	1219.5	1290.5
	P6	1249.73	±	115.25	1215.6	1285.8
	P12	1238.94	±	114.32	1203.1	1277.8

* Differences between P0 vs. P12, *p* = 0.041.
